# „Akuter“ Keratokonus?

**DOI:** 10.1007/s00347-021-01355-y

**Published:** 2021-03-10

**Authors:** Marlene Saßmannshausen, Martina C. Herwig-Carl, Frank G. Holz, Karin U. Loeffler

**Affiliations:** 1 Klinik für Augenheilkunde, Universitätsklinikum Bonn, Ernst-Abbe-Str. 2, 53127 Bonn, Deutschland; 2 Sektion Ophthalmopathologie, Universitätsklinikum Bonn, Bonn, Deutschland

## Anamnese

Ein 32-jähriger Patient wurde der Klinik notfallmäßig vom behandelnden Augenarzt mit Verdacht auf einen akuten Keratokonus (KK) zugewiesen. Der Patient berichtete, eine „Blase“ am rechten Auge bemerkt zu haben. Schmerzen oder eine akute Sehminderung wurden verneint. Vor 11 Jahren war bei KK ein korneales Cross-Linking (cCXL) durchgeführt worden. Das Tragen von Kontaktlinsen wurde verneint. Allgemeinanamnestisch war der Patient gesund.

## Befund

In der spaltlampenmikroskopischen Untersuchung zeigte sich rechts eine zentrale Hornhauttrübung mit einer Descemetozele ohne Hinweis auf eine akute Hornhautperforation bei einem sonst unauffälligen ophthalmologischen Befund und einem bestkorrigierten Visus von 0,2. Wir versorgten den Patienten zunächst mit einer therapeutischen Kontaktlinse, lokaler antibiotischer Therapie und vereinbarten eine kurzfristige Wiedervorstellung. Der Patient stellte sich bereits in der nächsten Nacht mit akuten Schmerzen und einer Visusminderung auf 0,05 vor. Er gab an, gegen das rechte Auge gestoßen zu sein. Hier zeigte sich nun eine Hornhautperforation, sodass wir notfallmäßig eine Keratoplastik à chaud durchführten.

## Histopathologische Untersuchung

Zur weiteren Untersuchung wurde die trepanierte Wirtshornhaut in 4%igem Paraformaldehyd fixiert und histologisch aufgearbeitet. Makroskopisch zeigte sich ein weitgehend klares Hornhautscheibchen mit einer zentralen Trübung (2 × 2 mm) und darin gelegener Perforationsstelle (1 × 0,5 mm).

**Figure 1:**
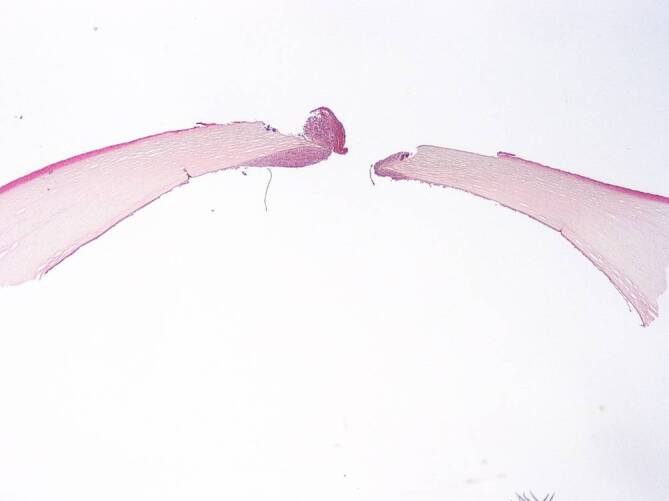

**Figure 2:**
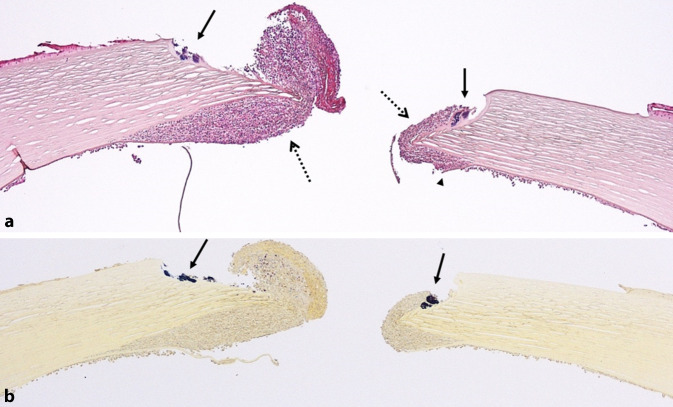

Im histologischen Übersichtsschnitt zeigt sich eine zentrale Hornhautperforation mit einem scharf begrenzten, aber dichten Rasen von Entzündungszellen (Abb. 1). Die Entzündungszellen dehnen sich teilweise zwischen der Descemet-Membran und der hintersten Stromalamelle aus (Abb. 2a). Das angrenzende Hornhautstroma selbst ist frei von Entzündungszellen. Im Bereich der Perforationsstelle lassen sich Konglomerate von grampositiven Bakterien identifizieren (Abb. 2b). Die Ränder der Descemet-Membran im Bereich der Perforationsstelle sind aufgesplittert (blauer Pfeil in Abb. 2a).

**Figure 3:**
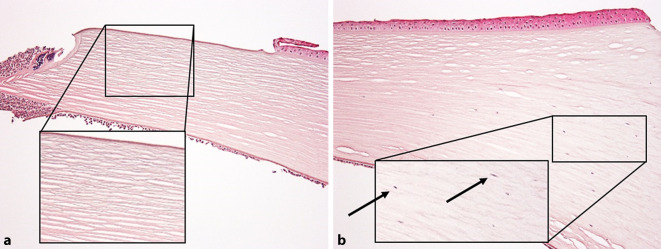

Zudem zeigt sich im Bereich der zentralen Hornhaut ein vollständiger Keratozytenverlust, während in der Peripherie noch einzelne Keratozyten sichtbar sind, die jedoch in ihrer Konfiguration verändert erscheinen (Abb. 3a, b).

## Diagnose und Verlauf

In der Zusammenschau der klinischen und histopathologischen Befunde stellten wir die Diagnose eines perforierten Hornhautulkus bei bekanntem KK mit einem ausgedehnten Keratozytenverlust nach cCXL. Im weiteren postoperativen Verlauf zeigte sich eine komplikationslose Heilung mit klarem Hornhauttransplantat und einer bestkorrigierten Sehschärfe von 0,5.

## Diskussion

Das cCXL ist eine mittlerweile breit verfügbare Therapieoption bei progredientem KK, das mittels lokaler Riboflavin-Gabe (Vitamin B_2_) und anschließender UVA-Bestrahlung der Hornhaut eine Quervernetzung der Kollagenfibrillen induziert und so zu einer Stabilisierung der Hornhaut führen soll [9]. Es handelt sich dabei um ein insgesamt risikoarmes Verfahren, mögliche Komplikationen in der postoperativen und auch langfristigen Betreuung von Patienten sind jedoch zu beachten. Postoperative Veränderungen und Komplikationen, neben einer Erosio als potenzielle Eintrittspforte für postoperative Entzündungen, sind postoperative stromale Trübungen und Narbenbildungen [6]. Eine mittelfristige Nebenwirkung, verursacht durch die zytotoxische Wirkung des cCXL selbst, ist u. a. der Verlust von Keratozyten. Regulär wird in diesem Zusammenhang eine vollständige Repopularisierung der Hornhaut mit Keratozyten bis zum 6. Monat (spätestens 12. Monat) nach der Behandlung angenommen [2, 4]. Eine zunehmende Anzahl von Arbeiten beschreibt jedoch das Phänomen eines persistierenden Keratozytenverlusts bei Patienten mit einem KK nach cCXL. Derzeit sind 11 im Rahmen einer perforierenden Keratoplastik entnommene Hornhäute mit Zustand nach cCXL bei bekanntem KK und persistierendem Keratoyztenverlust beschrieben [5, 7, 8]. Dazu zählt u. a. der hier beschriebene Fall [7].

Das Besondere an unserer Kasuistik ist zum einen der persistierende praktisch vollständige Keratozytenverlust in allen zentralen Stromaanteilen 11 Jahre nach dem cCXL, zum anderen aber auch die besondere Konfiguration von Erregern und Entzündungszellen im Bereich der Perforation. Offensichtlich lag hier weder klinisch noch histologisch ein akuter Keratokonus vor, da einerseits die Hornhaut kein diffuses massives Ödem aufwies und auch die feingewebliche Aufarbeitung nicht das Bild eines Hydrops mit eingerollten Rändern einer rupturierten Descemet-Membran, sondern ein infektiöses Geschehen mit Erregern und Entzündung zeigte. Im Gegensatz zu einem „normalen“ infektiösen Ulkus, bei dem meist eine ausgeprägte Entzündungszellinfiltration aller Hornhautschichten sowohl im Bereich der Hornhautperforationsstelle als auch etwas weiter entfernt vorliegt, zeigte sich in unserem Präparat eine atypische Ausprägung einer Entzündungszellinfiltration mit einer lokalen Begrenzung auf den Ulkusabhang und prädescemetale Anteile der Hornhaut bei gleichzeitig völlig azellulären und von den Entzündungszellen nicht betroffenen stromalen Hornhautlamellen.

Die Keratozyten selbst übernehmen regulär die Aufgabe der Regeneration und des Wiederaufbaus von geschädigten Hornhautstrukturen, und auch an der Rekrutierung von neutrophilen Granulozyten bei z. B. infektiösem Geschehen sind die Keratozyten maßgeblich beteiligt [1, 3]. Hier wird jedoch gezeigt, dass es im Rahmen eines CXL möglicherweise zu einer Veränderung der kornealen Matrixstruktur mit einem persistierenden Verlust von Keratozyten kommen kann und die daraus resultierende azelluläre Hornhaut wie eine Barriere gegenüber einer diffusen Ausbreitung von Entzündungszellen wirken könnte. Ebenso könnte durch den Keratozytenverlust die Rekrutierung von neutrophilen Granulozyten in die Hornhaut beeinträchtigt sein. Vier Wochen vor Vorstellung in unserer Klinik zeigte sich beim Augenarzt bei einer Sehschärfe von 0,2 eine zentrale Hornhautnarbe mit einem beginnenden Substanzdefekt, der antibiotisch bereits antherapiert wurde. Naheliegend ist, dass der vermutlich schon vorbestehende Keratozytenverlust über lange Zeit komplikationslos blieb, jedoch mit Auftreten eines oberflächlichen Hornhauttraumas mit nachfolgender Infektion eine akute Hornhautperforation begünstigte.

Zusammenfassend demonstriert dieser Fall eine relevante Komplikation eines länger zurückliegenden Eingriffes mit einem langfristig erhöhten Risiko für postoperative Komplikationen. Somit sollten bei Patienten mit Zustand nach cCXL langfristig regelmäßige augenärztliche Kontrollen erfolgen.

## Fazit für die Praxis

- Ein azelluläres Hornhautstroma kann auch Jahre nach einem kornealen Crosslinking (cCXL) noch persistieren.
- Ein azelluläres Hornhautstroma begünstigt möglicherweise die Perforation im Rahmen eines infektiösen Geschehens.
- Patienten mit Keratokonus und Zustand nach cCXL sollten langfristig regelmäßig augenärztlich untersucht werden.
